# The Regulation and Function of miR-21-FOXO3a-miR-34b/c Signaling in Breast Cancer

**DOI:** 10.3390/ijms16023148

**Published:** 2015-01-30

**Authors:** Xiangyan Liu, Jie Feng, Lili Tang, Liqiu Liao, Qing Xu, Shaihong Zhu

**Affiliations:** 1 Department of General Surgery, the Xiangya Hospital, Central South University, 138 Tongzipo Road, Changsha 410013, China; E-Mails: xiangyanliu@yahoo.com (X.L.); musicjie302@yahoo.com (L.L); 2 Department of Breast Surgery, the Xiangya Hospital, Central South University, 138 Tongzipo Road, Changsha 410013, China; E-Mail: lilitang474@yahoo.com; 3 Department of Nursing, Thomas Jefferson University, Philadelphia, PA 19107, USA; E-Mail: jie.feng@jefferson.edu; 4 Department of Pathology, Anatomy and Cell Biology, Thomas Jefferson University, Philadelphia, PA 19107, USA; E-Mail: qxwelcome@gmail.com

**Keywords:** miR-21, miR-34b/c, FOXO3a, breast cancer, miRNAs injection

## Abstract

Upregulation of miR-21 (microRNA-21) and downregulation of miR-34b/c have been found in breast cancer (BC). However, their regulation mechanism and function roles in BC have not been fully addressed. Here, we report that miR-21 levels were inversely correlated with miR-34b/c levels in BC. MiR-21 upregulation contributes to PTEN downregulation, which is beneficial for the activation of PI3K/AKT signaling. The activation of AKT phosphorylates FOXO3a, triggering relocalization of FOXO3a proteins from the nucleus to the cytoplasm. FOXO3a is a newly identified transcription factor responsible for miR-34b/c expression. Downregulation of nuclear FOXO3a decreased the expression levels of miR-34b and miR-34c in breast cancer cells, in which p53 was mutated. We also found upregulation of circulating miR-21 and downregulation of circulating miR-34b/c in BC patients’ serum. More importantly, we showed that systemic delivery of miR-34b/c or with anti-miR-21 significantly inhibited breast tumor growth *in vivo*. These results suggest that high circulating levels of miR-21 and low levels of miR-34b/c may provide potential biomarkers for BC diagnosis, and systemic delivery of miR-34b/c has potential as a therapeutic option for BC treatment.

## 1. Introduction

Breast cancer (BC) is the most common malignant disease in women in western countries and is a leading cause of cancer-related death among women worldwide [1]. The incidence rate of breast cancer is increasing dramatically in China in recent years. Although prognostic miRNAs expression signatures have been defined for some breast carcinomas [2,3,4], the underlying pathways regulating breast cancer aggressiveness remain poorly understood.

MiRNAs (microRNAs) are small endogenous non-coding RNAs that control target-gene expression at post-transcriptional levels. Many miRNAs are implicated as proto-oncogenes or as tumor suppressors and are aberrantly expressed in various cancer types including breast cancer. Among oncogenic miRNAs, miR-21 is one of the most commonly observed aberrant miRNAs in human cancers. MiR-21 dysregulation was first reported in breast cancer by Iorio [5]. MiR-21 has been reported to promote oncogenesis and progression of various carcinomas via direct targeting of tumor suppressing phosphatase and tensin homolog (PTEN) [6]. Recently, emerging evidences demonstrated that the miR-34 family (miR-34a, miR-34b, and miR-34c) are down-regulated and play an important role as a tumor suppressor in various types of cancers [7,8,9,10]. MiR-34a is encoded by its own transcript, whereas miR-34b and miR-34c share a common primary transcript. The miR-34 family directly targets/regulates many proteins involved in various biological processes, such as cell cycle, apoptosis, cell migration and metastasis [11]. Moreover, systemic delivery of miR-34a in a mouse model of lung tumor resulted in reduced tumor burden [12]. The past several years of studies have clearly shown that the miR-21 and miR-34 families are the master regulators of tumor biology. However, the regulation mechanisms and the therapeutic roles of miR-21 and miR-34b/c in breast cancer have not yet been fully clarified. In our study, we demonstrated that (i) miR-21 regulated miR-34b/c through affecting PI3K/AKT/FOXO3a signaling; (ii) circulating miR-21 and miR-34b/c have clinical implication for providing novel biomarkers for BC diagnosis; and (iii) targeting miR-21and/or restoring miR-34b/c may possess therapeutic applications for BC treatment in the future.

## 2. Results and Discussion

### 2.1. Expression of MiR-21 and MiR-34b/c in Breast Cancer Cell Lines, Clinical Specimens, and Serum Samples

**Figure 1:**
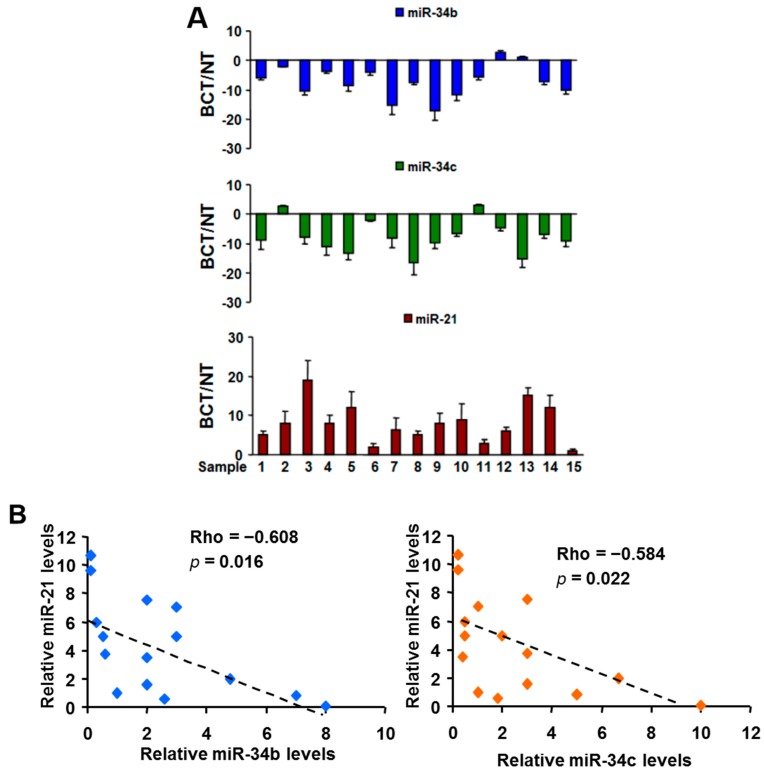

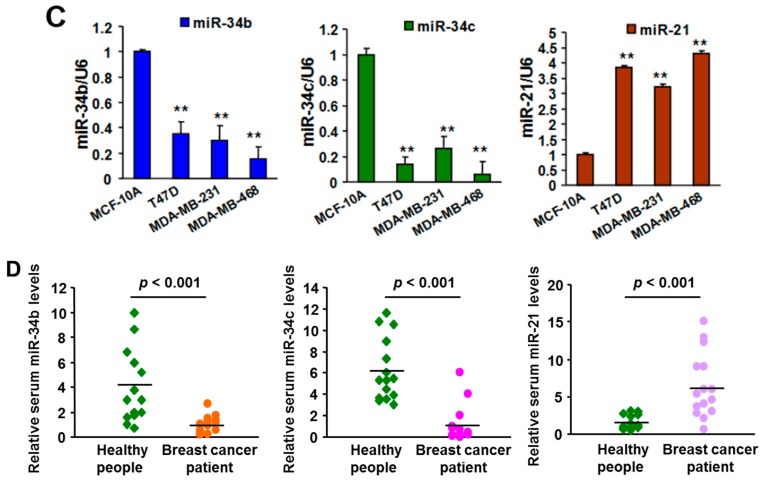
MiR-21 was upregulated and miR34b/c was down-regulated in breast cancer cells, tissues, and serums. (**A**) Relative expression levels of miR-21, miR-34b and miR-34c in 15 paired of breast cancer tumors (BCT) and non-malignant adjacent tissues (NT) were determined by RT-qPCR assay, and normalized to the U6 levels; (**B**) Scatter plots of the Spearman correlation coefficient (rho) corresponding to an inverted monotonic trend between relative miR-21, and miR-34b, or miR-34c levels in breast cancer tumors; (**C**) Levels of miR-21, miR-34b and miR-34 in MCF-10A, T47D, MDA-MB-231, and MDA-MB-468 cells were determined by RT-qPCR assay, and normalized to the U6 levels; and (**D**) Serum levels of miR-21, miR-34b and miR-34 in breast cancer patients and healthy people were analyzed by Taqman RT-qPCR assay. All results represent the mean ± SD from three independent experiments. ** indicate significant difference at *p* < 0.01, respectively.

We first conducted a miRNA array analysis to investigate the miRNAs expression profiles in three paired breast carcinoma tissues (BCT) and non-malignant adjacent tissues (NT). We identified several of the most significantly up- and down-regulated miRNAs that have been previously implicated in breast cancer, including miR-21 (up-regulated) and miR-34b/c (down-regulated). We subsequently validated this finding by quantitative RT-PCR (RT-qPCR) for miR-21, miR-34b, and miR-34c in an independent group of 15 paired BCT and NT (see Figure 1A). Notably, Spearman’s rank correlation analysis showed that the miR-21 level was inversely correlated with miR-34b (correlation = −0.608, *p* = 0.016, *n* = 15) and miR-34c (correlation = −0.584, *p* = 0.022, *n* = 15) levels in 15 breast cancer specimens (see Figure 1B). Next, we examined the expression levels of these miRNAs in three human BC cell lines and one immortalized breast epithelial cell line, MCF-10A. Consistent with the data obtained from breast tissue specimens, miR-21 was significantly up-regulated, and miR-34b/c was dramatically down-regulated in all three BC cell lines compared with MCF-10A (see Figure 1C). More interestingly, we found that levels of circulating miR-21 were higher, and circulating miR-34b/c were lower in BC patients’ serum than in serum from healthy controls (see Figure 1D), indicating that high circulating miR-21 and low miR-34b/c may serve as potential biomarkers for BC diagnosis.

### 2.2. Functions of MiR-21 and MiR-34b/c in Breast Cancer Cells

**Figure 2:**
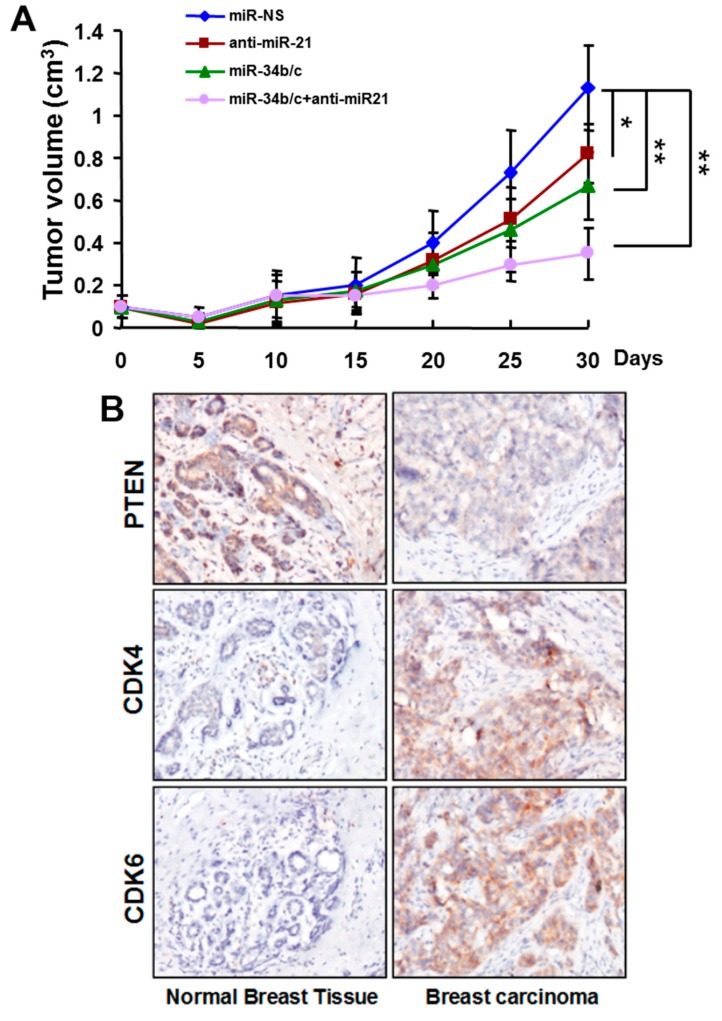

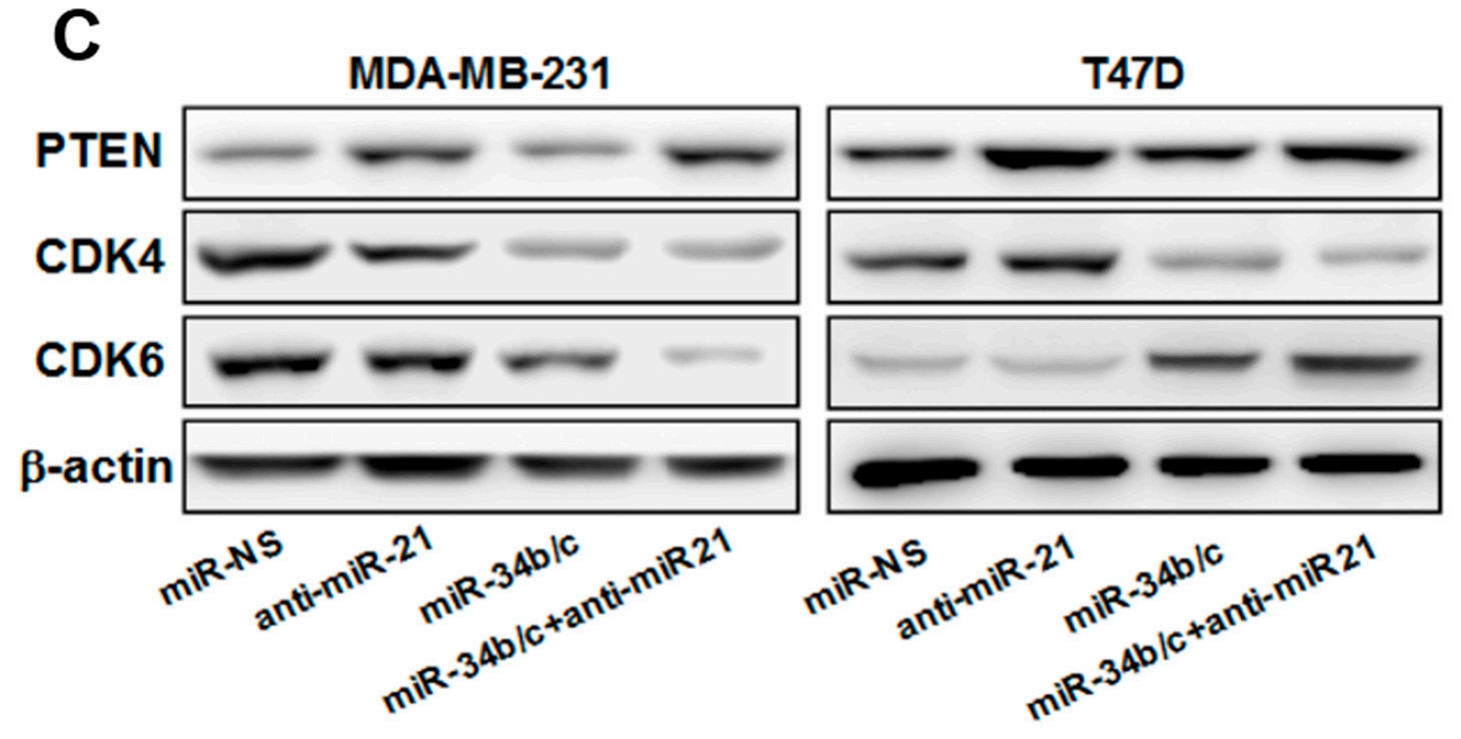
Functional roles of miR-21 and miR-34b/c in breast cancer. (**A**) MDA-MB-231 cells stable-expressing non-sense sequence (NS), anti-miR-21 and/or miR-34b/c were subcutaneously injected into immunodeficient mice. Tumor volumes were measured every five days. Xenografts were removed 30 days after implantation. Tumor volumes were obtained and presented (mean ± SEM; *n* = 8). * *p* < 0.05, ** *p* < 0.01; (**B**) Immunohistochemical (IHC) staining of PTEN, CDK4, and CDK6 in representative breast carcinoma tumors and non-malignant normal adjacent tissues. (×200 magnification); and (**C**) T47D and MDA-MB-231 cells were infected with lentivirus carrying non-sense sequence (NS), anti-miR-21 and/or miR-34b/c. The expression levels of PTEN, CDK4, and CDK6 in these cells were analyzed by Western blot.

To investigate the biological functions of miR-21, miR-34b, and miR-34c in BC cells, we used lentiviral vectors to establish T47D and MDA-MB-231 cell lines stably expressing non-sense sequence miR-NS, anti-miR-21, miR-34b/c, or triple expressing anti-miR-21 and miR-34b/c. We used these four miRNAs-stably expressing BC cell lines to perform *in vitro* and *in vivo* experiments, including cell growth, colony formation, cell migration, and tumor growth. The results showed that down-regulation of miR-21 and/or up-regulation of miR-34b/c led to a reduction in cellular proliferation, and migratory capacity, and impaired the ability of colony formation of both BC cell lines (see Supplementary Figure S1). We next examined the role for miR-21 and miR-34b/c *in vivo* by subcutaneous injection of miRNAs-stable expressing cells into nude mice. Within 30 days after injection, we found that miR-21 inhibition and/or miR-34b/c restoration resulted in substantial reduction of tumor growth comparing with control groups (see Figure 2A). Notably, among all treatment groups, the triple miRNAs-expressing groups exhibited the strongest cell proliferation, colony formation, cell migration, and tumor growth inhibition effect on BC cell lines.

To explore the mechanism of growth inhibition induced by anti-miR-21, and/or miR-34/c in BC, we searched a list of published or potential targets of miR-21 and miR-34b/c. We identified PTEN, a well known target of miR-21, and CDK4/6, two important targeted of miR-34b/c [13,14]. These proteins were crucial in miR-21- and miR-34b/c-mediated BC cellular function. We first evaluated the expression levels of PTEN, CDK4 and CDK6 in ten pairs of BC specimens by Western blot and immunohistochemical (IHC) staining. The results revealed that the expression of PTEN was down-regulated, and both CDK4 and CDK6 expression were up-regulated in breast tumor tissues and cancer cell lines compared with non-malignant adjacent tissues and MCF-10A, respectively (see Figure 2B and Supplementary Figure S2). Overexpression of anti-miR-21 increased PTEN expression, and restoration of miR-34b/c expression repressed CDK4/6 expression in BC cell lines (see Figure 2C).

### 2.3. MiR-21 Regulates MiR-34b/c Expression via PTEN/AKT/FOXO3a Signaling Pathway

**Figure 3:**
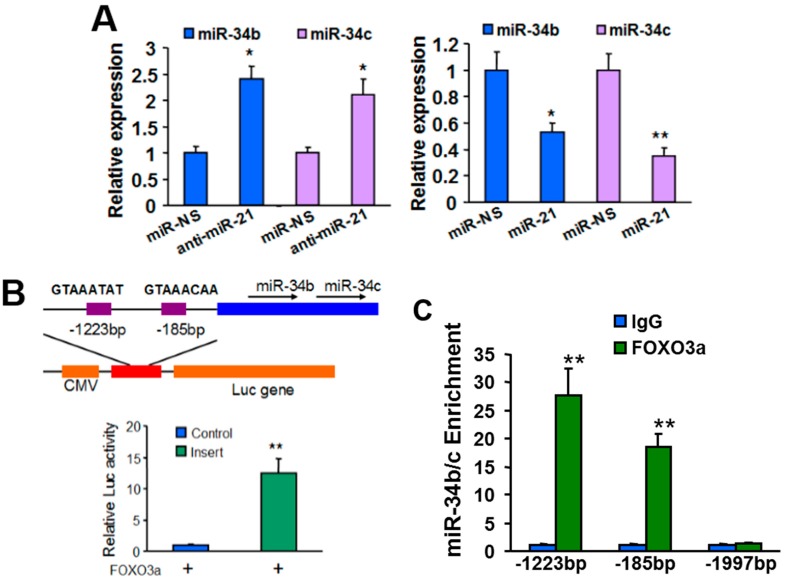

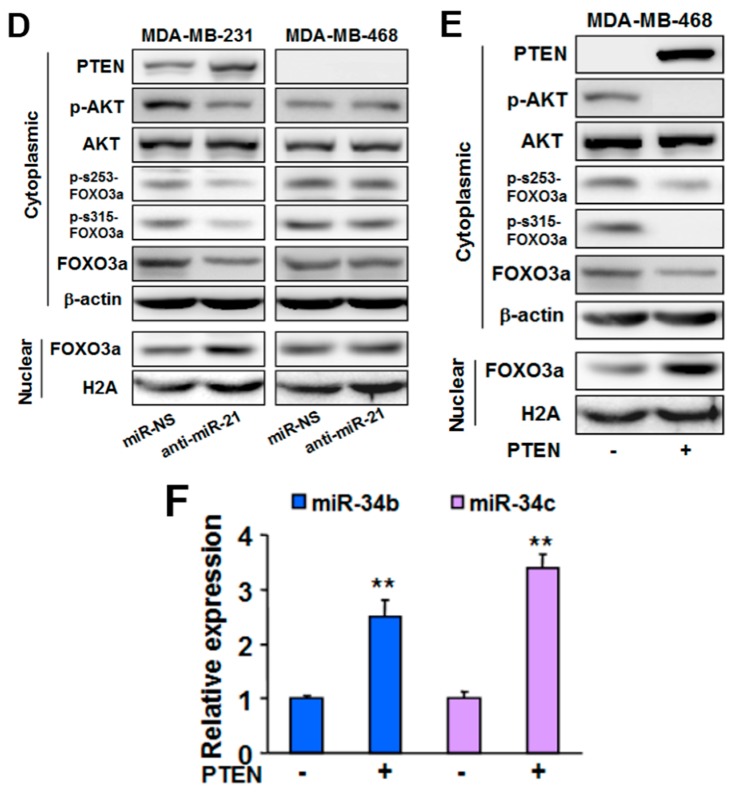
MiR-21 regulated miR-34b/c expression through AKT/PTEN/FOXO3a signaling. (**A**) MCF-10A cells were transfected with miR-NS, miR-21, or anti-miR-21 for 24 h. The expression levels of miR-34b and miR-34c were analyzed by RT-qPCR and normalized to the U6 levels; (**B**) Schematic diagrams show the potential FOXO3a binding sites of the miR-34b/c promoter and the construction of miR-34b/c promoter luciferase reporter. Boxes indicate the distribution of two putative FOXO3a binding sites. Sequences of the consensus FOXO3a-binding sites are also shown. The reporter plasmid was co-transfected with FOXO3a expression plasmid and β-gal plasmid. The luciferase activities were normalized to the activity of β-gal, and values shown are normalized to the value of pGL-3 basic construct; (**C**) MCF-10A cells were infected with lentivirus carrying FOXO3a or empty vector (Control). Cross-linked chromatin was immunoprecipitated with antibodies specific for FOXO3a and IgG. Purified DNA was analyzed by RT-qPCR with three specific primer pairs as indicated in the Figure. The primers located in −1997bp were used as a negative control; (**D**) MDA-MB-231 and MDA-MB-468 cells were infected with lentivirus carrying anti-miR-21 or miR-NS. Western blot analysis of nuclear levels of FOXO3a and cytoplasmic levels of PTEN, p-AKT, AKT, p-s253-FOXO3a, and p-s315-FOXO3a, respectively. H2A and β-actin were used as loading control; and (**E**,**F**) MDA-MB-468 cells were infected with lentivirus carrying PTEN or non-sense sequence. Western blot analysis of nuclear levels of FOXO3a and cytoplasmic levels of PTEN, p-AKT, AKT, p-s253-FOXO3a, and p-s315-FOXO3a in these cells, respectively, (**E**) or RT-qPCR analysis of miR-34b and miR-34c levels (**F**). Experiments were performed in triplicate and each was repeated three times. * *p* < 0.05, ** *p* < 0.01.

Interestingly, we found that miR-21 overexpression decreased the expression levels of miR-34b/c; while miR-21 knockdown enhanced miR-34b/c expression (see Figure 3A). To understand the molecular mechanism of how miR-21 regulates miR-34b/c expression, we searched for transcription factors involved in miR-34b/c expression. MiR-34b and miR-34c are processed from the same pre-miRNA (BC021736), indicating that miR-34b and miR-34c are regulated by the same transcription factor. FOXO3a was reported to be responsible for regulation of miR-34b/c expression [15]. To verify this conclusion in BC cell lines, we first constructed a luciferase reporter plasmid containing putative FOXO3a binding sites in the promoter region of BC021736. We observed that FOXO3a overexpression significantly enhanced the luciferase activity from the reporter construct (see Figure 3B). Chromatin immunoprecipitation-qPCR (ChIP-qPCR) results showed that the FOXO3a occupancy of the BC021736 promoter was markedly higher compared to the IgG background signals and negative control signals (see Figure 3C). The expression levels of miR-34b and miR-34c were enhanced by FOXO3a overexpression, but suppressed by siRNA-mediated knockdown of FOXO3a expression in MCF-10a cells (see Supplementary Figure S3A). Taken together, these results suggest that miR-34b and miR-34c are regulated by FOXO3a at transcriptional levels. We observed that forced expression of FOXO3a in miR-21 stable-expressing cell lines abolished miR-21-treated miR-34b/c down-regulation (see Supplementary Figure S3B), indicating FOXO3a is involved in miR-21 regulated miR-34b/c expression.

Given that phosphorylation of FOXO3a by oncogenic kinases, such as AKT or ERK results in FOXO3a translocation from nucleus to cytoplasm and subsequent degradation [16], and PTEN is a well-characterized AKT pathway suppressor, we hypothesized that miR-21 regulated the activity of FOXO3a through the AKT/PTEN signaling pathway. To verify this hypothesis, MDA-MB-231 (PTEN+/+) and MDA-MB-468 (PTEN−/−) cells were transfected with anti-miR-Scr or anti-miR-21. We found that suppressing miR-21 enhanced levels of PTEN and nuclear levels of FOXO3a, and decreased levels of p-AKT, p-s253-FoxO3a and p-s315-FoxO3a in MDA-MB-231 with wild-type PTEN expression, but not in MDA-MB-468 with PTEN depletion (see Figure 3D). MiR-21 knockdown induced nuclear FOXO3a up-regulation leading to a robust increase of FOXO3a occupancy at the promoter of BC021736 in MDA-MB-231 cells (see Figure 3C). Moreover, forced expression of PTEN in MDA-MB-468 can mimic the effect of knockdown of miR-21 in MDA-MB-231 cell (see Figure 3E,F). Taken together, our results suggested that miR-21 regulates miR-34b/c expression via the PTEN/AKT/FOXO3a signaling pathway.

### 2.4. Systemic Delivery of MiR-34b/c Inhibits the Growth of Breast Tumors

**Figure 4:**
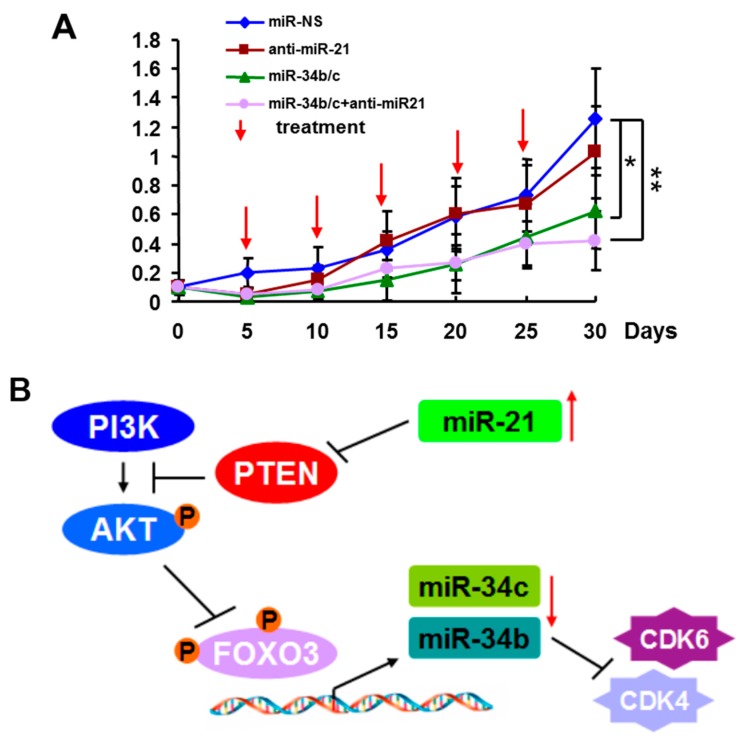
Systematic delivery of miR-34b/c or with anti-miR-21 inhibited breast tumor growth. (**A**) MDA-MB-231 cells were subcutaneously injected into immunodeficient mice. Tumor volumes were measured every five days. At day five after tumor implantation, the mice were treated with formulated miR-Scr, anti-miR-21, miR-34b/c, or anti-miR-21 and miR-34b/c combination by intravenous (i.v.) tail vein every five days. Tumor volumes were measured every five days and presented. (mean ± SEM; *n* = 8). * *p* < 0.05, ** *p* < 0.01; and (**B**) A model of the miR-21-FOXO3a-miR-34b/c signaling in breast cancer. Oncogenic miR-21 up-regulation promoted PI3K/AKT signaling activation through directly inhibiting PTEN expression, a suppressor of PI3K/AKT. The activation of AKT phosphorylates FOXO3a resulting in relocalization of FOXO3a proteins from nucleus to the cytoplasm. Nuclear FOXO3a down-regulation reduced the binding efficiency of FOXO3a in the promoter of miR-34b/c leading to decreases in the expression levels of miR-34b and miR-34c in cells.

To investigate the therapeutic effect of miR-21 and/or miR-34b/c on breast tumors, we administrated formulated miRNAs by intravenous (i.v.) tail vein injection to BALB/c athymic nude mice bearing pre-established tumors. MDA-MB-231 cells were subcutaneously injected into the lower back of nude mice. On day five after xenograft implantation, tumor-bearing mice received i.v. tail vein injection of formulated miR-Scr, anti-miR-21, miR-34b/c, or anti-miR-21 and miR-34b/c combination. Each dose contained 20 μg of miRNAs, which equals 1 mg/kg per mouse. Mice received formulated miRNAs injection every five days for a total of five times, and tumor volumes were measured each time before injection. As shown in Figure 4A, i.v. delivery of miR-34b/c, or anti-miR-21 and miR-34b/c combination dramatically reduced breast tumor growth in mice, and triple miRNAs delivery resulted in the greatest tumor growth suppression. However, the different tumor weight between anti-miR-21 injection and miR-Scr injection did not reach statistical significance in this study. These results indicate that targeting miR-21 and/or restoration of miR-34b/c exerts potential therapeutic efficacy against breast tumors.

### 2.5. Discussion

#### 2.5.1. MiR-21 and MiR-34b/c Expression in Breast Cancer

A large-scale study of 540 human samples firstly demonstrated that miR-21 is the only miRNA that is up-regulated in six human cancers [17]. Years of studies demonstrated that the miR-34 family is down-regulated in most cancer types studied. Consistent with these observations, in the present study, we found that miR-21 was over-expressed and miR-34b/c was decreased in breast carcinoma of a Chinese population. Because serum or plasma samples are more feasible than tumor tissues in clinical practice, circulating miRNAs are highlighted as potential diagnostic markers for human cancer. Circulating miR-21 and miR-34a were reported to be up-regulated and down-regulated in breast cancer patients [18,19], respectively, however, there has been no information related to circulating miR-34b and miR-34c in breast cancer patients. Our findings not only confirmed that serum levels of miR-21 in breast cancer patients are higher than in healthy controls, but also showed for the first time that circulating miR-34b and miR-34c were significantly down-regulated in serum from breast cancer patients, indicating that miR-21 and miR-34b/c are candidate serum biomarkers for breast cancer.

#### 2.5.2. Mechanism of MiR-21 Regulation of MiR-34b/c Expression

It has been reported that the miR-34 family is regulated by p53 in p53 wild-type cells. In breast cancer cell lines (MDA-MB-231, MDA-MB-468, and T47D), the expression of miR-34b/c is independent of p53 regulation due to the inactivating mutations of p53. We found that miR-21 levels were inversely correlated with miR-34b and miR-34c levels, and induction of miR-34b/c in response to miR-21 down-regulation occur in breast cancer cells raising the possibility that miR-34b/c is regulated by other transcription factor in cancer cells. AKT phosphorylates FOXO3a triggering the rapid relocalization of FOXO3a proteins from nucleus to the cytoplasm, leading to repression of FOXO3a transcriptional activity [20,21]. We showed that miR-21 inhibition led to enhancing PTEN expression, and the latter inhibited the activity of AKT. Dephosphorylation of FOXO3a due to AKT inactivation, promotes FOXO3a nuclear translocation, resulting in enhanced FOXO3a-driven miR-34b/c upregulation. Although it has been shown that miRNAs can regulate other miRNAs via a direct interaction [22], we propose that miRNA-mediated miRNA expression through affecting transcription factor activity is more prevalent than miRNA-miRNA interaction in cells, since signal transduction has greater flexibility in regulation than the restrict requirements of miRNAs sequence binding.

#### 2.5.3. Functions of MiR-21 and MiR-34b/c in Breast Cancer

It is well established that miR-21 acts as an oncogene and miR-34b/c as tumor suppressors in human cancer [23,24]. Recently, miRNAs have emerged as therapeutic options to treat cancer. The advantages of miRNAs-based treatment are obvious. Firstly, miRNAs can regulate a broad set of genes simultaneously. Secondly, miRNAs can also affect tumor-promoting stromal cells to modulate the tumor microenvironment. Thirdly, miRNAs showed reduced immune response and low toxicity when compared with lentivirus- or protein-based gene therapy [25]. The effect of systemic delivery of anti-miR-21 and/or miR-34b/c on breast tumors has never been evaluated. Our results demonstrate that co-injection of miR-34b/c with anti-miR-21 achieved stronger anti-tumor effect than miR-34b/c or anti-miR-21 alone-treatment. This finding further supports the notion that application of miRNAs as a therapeutic option for cancer treatment, and multiple miRNAs administration may elicit better therapeutic effect than single miRNA. Further studies focusing on improving miRNAs stability and delivery efficiency are crucial for clinical use of miRNA-based therapy.

## 3. Experimental Section

### 3.1. Antibodies, Cell Lines, and Reagents 

Antibodies were as follows: PTEN, CDK4, CDK6, AKT, p-AKT, FOXO3a, p-s253-FOXO3a, and p-s315-FOXO3a were purchased from Cell Signaling Technology (Beverly, MA, USA). β-actin and H2A were obtained from Santa Cruz Biotechnonlgy (Santa Cruz, CA, USA). siRNA-targeting FOXO3a oligonucleotides was purchased from Thermo Scientific (Hudson, NH, USA) as specific oligo pools. Lentiviral plasmids expressing FOXO3a and PTEN were obtained from GeneCopoeia (Rockville, MD, USA), and anti-miR-21, miR-34b, and miR-34c were from (Open Biosystems, Pittsburgh, PA, USA), respectively. MCF-10A, T47D, MDA-MB-231 and MDA-MB-468 cells were purchased from ATCC (American Type Culture Collection, Manassas, VA, USA). Cells were maintained in 5% CO_2_ incubator at 37 °C.

### 3.2. Patients and Tissues Samples

A total of 15 pairs of breast carcinoma tumors and adjacent normal tissues were obtained from Xiangya Hospital, Changsha, China. None of these patients received preoperative chemotherapy or radiotherapy before the surgery. EDTA-Blood samples were collected from healthy donors or breast cancer patients and processed for plasma isolation immediately. All samples were frozen and stored at −80 °C. Written informed consents were obtained from all patients in this study. This study was approved by the Ethics Committee of the Medical College of Central South University and Xiangya Hospital.

### 3.3. Quantitative RT-PCR

The expression levels of miRNAs were analyzed using Taqman MicroRNA Assay Kits (Applied Biosystems, Foster City, CA, USA) specific for hsa-miR-21, hsa-miR-34b and has-miR-34c. All experiments were performed in triplicate.

### 3.4. Cell Migration Assay

MDA-MB-231 or T47D cells stably-expressing non-sense sequence (NS), anti-miR-21 and/or miR-34b/c as indicated were plated in the top chamber. After incubation, the top chambers were wiped with cotton wool to remove the non-migratory cells. The exterior sides were fixed with 100% cold methanol, stained with 0.1% crystal violet, and air-dried. Cells were counted under a microscope.

### 3.5. Immunohistochemistry Assay

IHC was performed as previously described [26]. Tumor samples were fixed and processed by the paraffin-embedded method. Tissue sections were deparaffinized in xylene, and rehydrated in a graded ethanol series. After blocking with 5% BSA, slides were probed overnight at 4 °C with monoclonal antibody against CDK4, CKD6 or PTEN. Primary antibody was detected by biotinylated secondary antibody followed by avidin-biotin peroxidase complex and 3,3'-diaminobenzidine chromagene (Life Technologies, Gaithersburg, MD, USA).

### 3.6. Chromatin Immunoprecipitation-qPCR (ChIP-qPCR)

ChIP-qPCR assays were performed using ChIP Assay Kit (Qiagen, Chatsworth, CA, USA). Purified DNA immunoprecipitated by FOXO3a or IgG antibody was used as a template for quantitative-PCR analysis. Primer sequences used in this study are listed as following: −185 Fwd (Forward) 5'-AATCACCAAAAGCCCAGAAA-3', −185 Rev (Reverse) 5'-TTTTAAATGCCCACACAGAGG-3'; −1223 Fwd 5'-CCAAGATATGGTTTTATATTTCCTATGAG-3', −1223 Rev 5'-CTTGGCCTCTCACAGTGCTA-3'; −1997 Fwd 5'-CTGTAATCCCAGCTACTCA-3', −1997 Rev 5'-TTGGCTCACTGCAACCTC-3'.

### 3.7. Mouse Xenograft Models

BALB/c athymic nude mice were housed and maintained in a laminar airflow cabinet in a pathogen-free environment. MDA-MB-231 cells (1 × 10^6^) stably-expressing miR-NS, anti-miR-21, miR-34b/c, or miR-34b/c + anti-miR-21, were mixed with Growth Factor Reduced Matrigel (BD Lifesciences, Maryland, MD, USA) and injected subcutaneously into the lower back of each animal. Thirty days after injection, the mice were sacrificed by euthanasia and primary tumors were harvested from mice.

For miRNAs injection treatment, MDA-MB-231 cells (1 × 10^6^) were mixed with Growth Factor Reduced Matrigel and injected subcutaneously into the right flank of each animal. miRNAs were formulated with MaxSuppressor *in vivo* RNALancerII (Bioo Scientific, Austin, TX, USA) according to the manufacturer’s instructions. Each dose contained 20 μg of formulated oligo, which equals 1 mg/kg per mouse with an average weight of 20 g. Formulated miRNAs were intravenously (i.v.) by tail vein injected every 5 days starting on day 5 after tumor cells implantation.

### 3.8. Statistical Analysis

All results were analyzed using SPSS for windows version 13 (SPSS, Chicago, IL, USA). Quantitative variables were analyzed by *t*-test or ANOVA. The correlations were analyzed using Spearman’s rank test. Differences were considered significant with *p* less than 0.05.

## Supplementary Materials

Supplementary materials can be found at http://www.mdpi.com/1422-0067/16/02/3148/s1.
